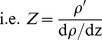# A Vertical Wall Dominated by *Acesta excavata* and *Neopycnodonte zibrowii*, Part of an Undersampled Group of Deep-Sea Habitats

**DOI:** 10.1371/journal.pone.0079917

**Published:** 2013-11-18

**Authors:** Mark P. Johnson, Martin White, Annette Wilson, Laura Würzberg, Enrico Schwabe, Helka Folch, A. Louise Allcock

**Affiliations:** 1 Ryan Institute and School of Natural Sciences, National University of Ireland Galway, Galway, Ireland; 2 Biocenter Grindel and Zoological Museum, University of Hamburg, Hamburg, Germany; 3 Bavarian State Collection of Zoology, Munich, Germany; 4 School of Biological Sciences, Queen’s University Belfast, Belfast, United Kingdom; Heriot-Watt University, United Kingdom

## Abstract

We describe a novel biotope at 633 to 762 m depth on a vertical wall in the Whittard Canyon, an extensive canyon system reaching from the shelf to the deep sea on Ireland’s continental margin. We explored this wall with an ROV and compiled a photomosaic of the habitat. The assemblage contributing to the biotope was dominated by large limid bivalves, *Acesta excavata* (mean shell height 10.4 cm), and deep-sea oysters, *Neopycnodonte zibrowii,* at high densities, particularly at overhangs. Mean density of *N. zibrowii* increased with depth, with densities of the most closely packed areas of *A. excavata* also increasing with depth. Other taxa associated with the assemblage included the solitary coral *Desmophyllum dianthus*, cerianthid anemones, comatulid crinoids, the trochid gastropod *Margarites* sp., the portunid crab *Bathynectes longispina* and small fish of the family Bythitidae. The scleractinian coral *Madrepora oculata,* the pencil urchin *Cidaris cidaris* and a species of *Epizoanthus* were also common. Prominent but less abundant species included the flytrap anemone *Actinoscyphia saginata,* the carrier crab *Paramola cuvieri,* and the fishes *Lepidion eques* and *Conger conger*. Observations of the hydrography of the canyon system identified that the upper 500 m was dominated by Eastern North Atlantic Water, with Mediterranean Outflow Water beneath it. The permanent thermocline is found between 600 and 1000 m depth, i.e., in the depth range of the vertical wall and the dense assemblage of filter feeders. Beam attenuation indicated nepheloid layers present in the canyon system with the greatest amounts of suspended material at the ROV dive site between 500 and 750 m. A cross-canyon CTD transect indicated the presence of internal waves between these depths. We hypothesise that internal waves concentrate suspended sediment at high concentrations at the foot of the vertical wall, possibly explaining the large size and high density of filter-feeding molluscs.

## Introduction

The continental margins, along with other parts of the deep oceans, still represent locations of discovery science [1], [2]. The novelty of deep-sea habitats is reflected by a habitat-discovery curve that does not yet have a plateau [3]. This relative lack of knowledge of deep-sea habitats is coupled with incompletely characterized, but important, ecosystem functions and services including nutrient recycling, carbon sequestration and nursery areas [4]. Sampling constraints have certainly restricted the description of deep-sea habitats. The vertical faces of canyon walls and other related habitats have only become accessible to survey with the availability of deep-water ROVs [5], [6].

The Whittard Canyon is one of the major submarine canyons along the Celtic margin, situated between the two main North Atlantic gyres. The region is an area of high primary production, with estimates of ca. 160 gC m^−2^ a^−1^ at the Goban Spur [7]. The Whittard Canyon floor has been found to be locally enriched in particulate organic carbon and phytodetritus (chl a) and labile lipids, suggesting high food quality in comparison to the open slope [8]. The NE Atlantic continental margin is characterized by a poleward flowing slope current, with typical long term mean flow in the vicinity of the Whittard Canyon of 5–10 cm s^−1^ [9] [10] [11]. The Celtic Sea region, which the Whittard Canyon fringes, is characterized by high barotropic tidal energy, with subsequent conversion to baroclinic internal tides (e.g., [12], [13]). The region is one where internal waves are generated by the residual flow over the rough margin topography (e.g., [14]).

In the results presented here, we describe a novel biotope from a vertical wall in the Whittard Canyon system: vertical surfaces and overhangs at depths between 633 and 762 m covered by the bivalve *Acesta excavata* (Fabricius, 1779) and the giant deep-sea oyster *Neopycnodonte zibrowii* Gofas, Salas & Taviani, in Wisshak et al., 2009. The term biotope describes the combination of a characteristic suite of species with an associated physical habitat [15]. A suite of species found together is referred to as an assemblage throughout the current study. The term assemblage is sometimes considered synonymous with community when describing the species found together in the same location; however, we use assemblage and follow the convention that the use of the term community implies that more is known about biological interactions between species.

*Acesta excavata* is found in the North Atlantic between Mauritania and Norway with scattered Mediterranean records; typical depths are between 200 and 800 m [16]. Outside of Norwegian fjords, *A. excavata* is considered to be a component of reefs of the cold-water coral *Lophelia pertusa*, whereas vertical walls in fjords are often covered by attached *A. excavata* [16]. *Neopycnodonte zibrowii* is not generally recognized as associated with *L. pertusa* habitats, although the oyster may occur in relatively close proximity to cold-water corals. Extensive vertical reefs of *N. zibrowii* have been reported from the canyons of the Bay of Biscay [5].

In the current study we describe the occurrence of an *A. excavata* -*N. zibrowii* biotope on a vertical wall. This adds to the known diversity of deep-sea habitats, contributing to the habitat-based framework for conservation planning [17]. We analyse quantitative biological and oceanographic measurements to understand more about the cross-habitat variations in the assemblage. This identifies some of the constraints that may be operating to shape the occurrence and extent of the habitat identified.

## Results

### Habitat Scope and Inhabitants

**Figure 1:**
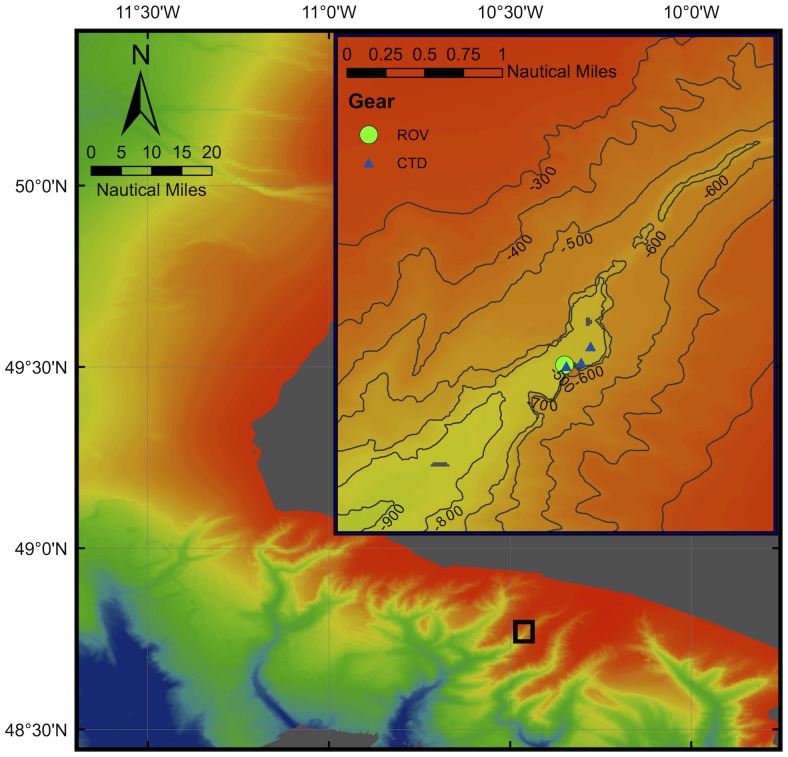
Location of the *A. excavata* -*N. zibrowii* biotope on the southern side of the surveyed canyon (green circle on inlay marked as ‘ROV’). Deeper waters have cooler colours.

On an ROV dive on 16^th^ April 2012, at 48.761°N, 10.461°W (Figure 1) we encountered a vertical wall extending from 631 m depth to 780 m depth. On this wall we identified the presence of large numbers of *Acesta excavata* on the vertical faces.

**Figure 2:**
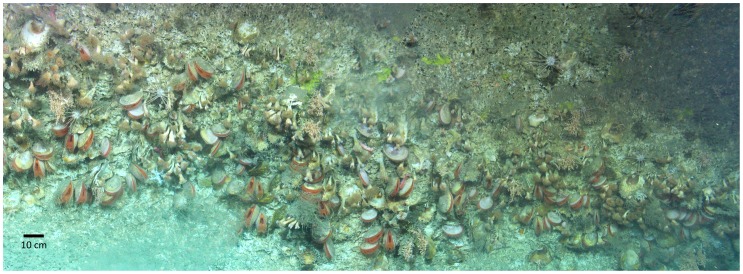
Photomosaic of *A. excavata* -*N. zibrowii* habitat at a depth of 666 m. Total area approximately 5^2^. A prominent *N. zibrowii* is visible in the top left of the image, with other oyster individuals embedded in the matrix of *A. excavata* and other species.

*Acesta excavata* and *Neopycnodonte zibrowii* were found together, mostly between depths of 633 m and 762 m, with particularly high densities in the vicinity of small overhangs (Figure 2). Nine taxa apart from the bivalves were identifiable in more than 10% of photographs. The two most abundant species were the scleractinian corals *Desmophyllum dianthus* and *Madrepora oculata* (Table 1). Comatulid feather stars were also relatively abundant, although the resolution of the photographs was sufficient neither to identify the feather stars to species level nor to conclude that they comprised a single species. In order of decreasing abundance was a species of small pink fish of the family Bythitidae living in the cryptic habitat created by the bivalves and corals, a pink tube anemone of the family Cerianthidae, a trochid gastropod *Margarites* sp., a portunid crab *Bathynectes longispina*, and the pencil urchin *Cidaris cidaris.* A species of *Epizoanthus* was also very abundant, being present on 24% of oyster shells.

**Table 1:** Correlations between taxa counted in photographs and the densities of *A. excavata* and *N. zibrowii*.

Taxon	Mean density (m^−2^) ±SE	Correlation with *A. excavata*	Correlation with *N. zibrowii*
*Desmophyllum dianthus*	24.0±4.42	0.552**	0.438**
*Madrepora oculata*	3.10±0.397	0.214	0.112
Comatulida sp(p).	2.23±0.629	0.619**	0.348
Bythitidae sp.	0.50±0.137	0.372	0.564*
Cerianthidae sp.	0.45±0.163	0.482*	0.594**
*Cidaris cidaris*	0.25±0.071	−0.001	−0.190
*Margarites* sp.	0.16±0.062	0.461*	0.473*
*Bathynectes longispina*	0.12±0.058	0.492*	0.505*

Results are only shown for taxa recorded in over 10% of photographs; other taxa were seen in fewer than 10% of photographs. Significant correlations are indicated using *p<0.05, **p<0.01, ***p<0.001. Other taxa were seen in less than 10% of photographs and are listed in the results section.

**Figure 3:**
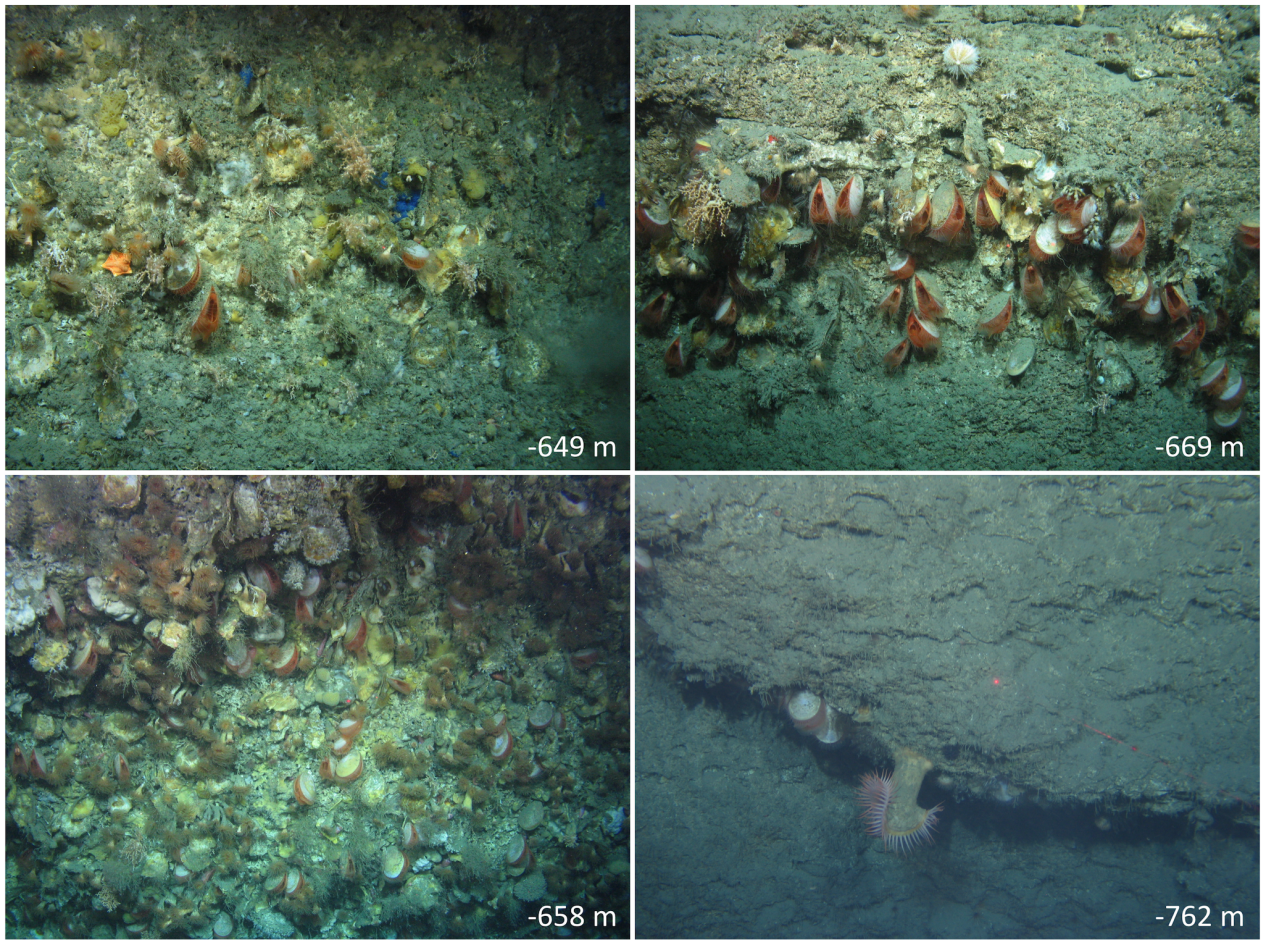
Images from different depths on the wall showing qualitative differences with depth, including more sponge cover at the shallowest depth, increased *A. excavata* and *N. zibrowii* with depth until the deepest, low biomass, section is reached.

Other species were present in less than 10% of the photographs. Some of these species were large and conspicuous despite occuring infrequently, for example, the bright orange flytrap anemone *Actinoscypha saginata* (Figure 3, bottom right), the carrier crab *Paramola cuvieri, Lepidion eques*, a characteristic fish species of deep waters in the region [18], and a conger eel, *Conger conger*, which was encountered at a depth of 681 m on the wall. Other crustaceans present in the photographs were squat lobsters Munidae sp., unidentified caridean shrimps and the euphausiid *Meganyctiphanes norvegica.* Apart from the crinoids and pencil urchin mentioned above, echinoderms in the photographs included four species of starfish. These comprised two poraniids, one almost certainly *Poranius pulvillus*, the other unidentified, a species of *Ceramaster* (Figure 3, upper left), and a stichastrellid, probably *Stichastrella rosea.* A second urchin species *Echinus* sp. (Figure 3, upper right) was also present. Other cnidarian species identified were the antipatharian coral *Stichopathes* sp. of which just two specimens were seen, a second species of zoanthid, *Parazoanthus anguicomis,* which was identified on a very small number of oyster shells, and the athecate hydrozoan *Tubularia indivisa*, which was more prevalent in shallower depths. Polychaetes are likely abundant in this cryptic habitat but are rarely visible in photographs. Two photographs capture Sabellidae sp. with their feeding tentacles displayed. This habitat is highly suitable for sabellids but it is likely that the turbulence caused by the ROV thrusters causes them to withdraw their characteristic tentacles making them hard to locate on photographs. Bryozoa were encountered relatively frequently but they were usually small and it was mostly not possible to identify them nor to elucidate the number of species present. However, a reticulate species of *Reteporella*, possibly *R. incognita*, was present, as was a cyclostome species that was probably *Tervia irregularis*. Sheet encrusting cheilostomes were occasionally present on oyster shells but could not be identified further. Distinctive sponges included the hadromerid *Weberella bursa*, the poecilosclerid *Mycale lingua* and an unidentified species of Tetractinellida. Two further sponge species were also tentatively identified from the upper part of the wall but the difficulty of sampling in overhang areas means that no voucher specimens were collected for confirmation. Nevertheless the blue sponge (Figure 3, upper left) could be *Hymedesmia curvichela* sensu [19] (not *Hymedesmia curvichela* Lundbeck, 1910) which we encountered (and collected) on a previous cruise to a canyon system north of the Porcupine Seabight. The bright yellow crust (also Figure 3, upper left) is likely *Hexadella dedritifera* Topsent, 1913, although this has recently been shown to be a species complex [20].

### Assemblage Structure

**Figure 4:**
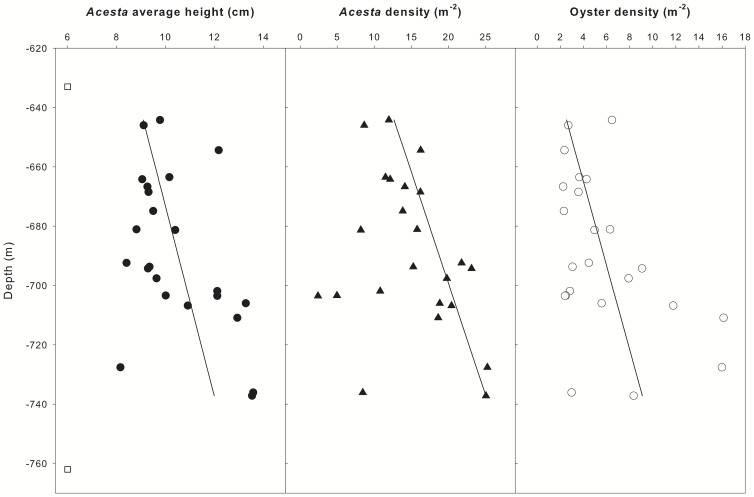
Variations in mean *A. excavata* shell height, *A. excavata* density and *N. zibrowii* density as a function of depth. The range of depths where *A. excavata* shells were observed on the wall was from 633 to 762 m (open square symbols). Lines are fitted linear regressions except for the panel displaying *A. excavata* densities where the line is a quantile regression estimating the position of the third quartile (0.75). Quantile regression coefficients were significantly different from zero when tested using bootstrap estimates of SE.

Size and density measurements in photographs were related to depth (Figure 4). The average height of *A. excavata* shells tended to increase with depth (F_1,20_ = 5.41, p<0.05, r^2^ 21%). The overall size distribution of measured shells was generally symmetrical around the mean of 10.5 cm (SE 0.37), with no obvious additional size classes. A linear regression of *A. excavata* density with depth was not significant; however, the highest densities in 20 m depth bands seemed to increase with depth. This observation was supported by quantile regression: coefficients for the 75% quantile were significant, indicating that upper density limits for *A. excavata* increased with depth. The maximum *A. excavata* density recorded was 25.2 m^−2^. *Neopycnodonte zibrowii* also occurred at higher densities with increasing depth (F_1,20_ = 4.6, p<0.05, r^2^ 19%). The maximum oyster density observed was 16.1 m^−2^. The patterns of increasing density with depth ended abruptly, with the deepest individuals found at low densities (values not directly estimated due to reflections of the laser sights off the substrate).

Correlations between species in the assemblage suggest common responses to environmental gradients and/or a biological interaction. *Acesta excavata* and *N. zibrowii* densities were positively correlated (r = 0.662, p<0.01). Of the nine identifiable taxa frequently observed in photographs, six were positively correlated with *A. excavata* and/or oyster densities: *Desmophyllum dianthus*, Cerianthidae sp, Comatulida sp(p)., Bythitidae sp., *Bathynectes longispina* and *Margarites* sp. (Table 1). The densities of two conspicuous species, the coral *Madrepora oculata* and the urchin *C. cidaris,* had no associations with *A. excavata* or *N. zibrowii*. Because of the nature of zoanthid colonies and the difficulties in identifying discrete colonies, it was not possible to test whether these were positively correlated with bivalve densities.

Other aspects of the habitat varied with depth. Most strikingly, the deepest part of the cliff had few *A. excavata* and the rest of the rock surface was relatively bare. This contrasts with shallower areas, where *A. excavata* was frequent, as were mobile species and colonies of corals, bryozoans, hydroids and sponges (Figure 3).

### Hydrography

**Figure 5:**
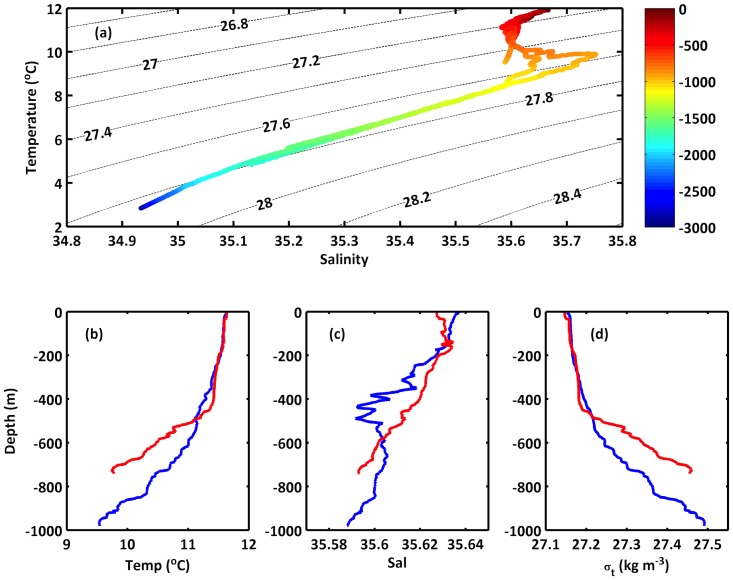
Water mass properties in the Whittard Canyon. A. Temperature-salinity plots (CTD casts at 750 m, 1000 m, 1820 m, 3100 m). Isopycnals indicate potential density, σ_t_ (kg m^−3^) and the colourbar indicates depth (m). B. Temperature (°C) profiles at 750 m (dash line) and 1000 m (solid line). C. Salinity profiles at 750 m and 1000 m. D. Density,σ_t_ (kg m^−3^) profiles at 750 m and 1000 m.

The water masses at the Whittard Canyon region are dominated by Eastern North Atlantic Water (ENAW) in the upper 500 m (Figure 5A) with ranges of 8< T <18°C, 35.2< S <36.7, and density, σ_t_ = 27–27.2 kg m^−3^. Below this, lies Mediterranean Outflow Water (MOW, 2.6< T <11°C, 35< S <36.2, with a core centred at σ_t_ = 27.5 kg m^−3^), found principally off slope, although the influence of MOW can be seen extending into the canyon branches. Labrador Sea Water (LSW) is found at intermediate depths with a core between 1900–2000 m. Vertical profiles of temperature, salinity and density, σ_t_ (Figure 5B–D) indicated a weakly stratified surface layer down to 400 m; the spring timing of the survey had not permitted full seasonal stratification to be developed. Between 600–1000 m evidence of the permanent thermocline can be seen, with strong stratification of the water column in both profiles. The ROV dive site (water depth 750 m), and the depths of the wall where high densities of suspension feeders were found (640–740 m), are located within this permanent thermocline.

**Figure 6:**
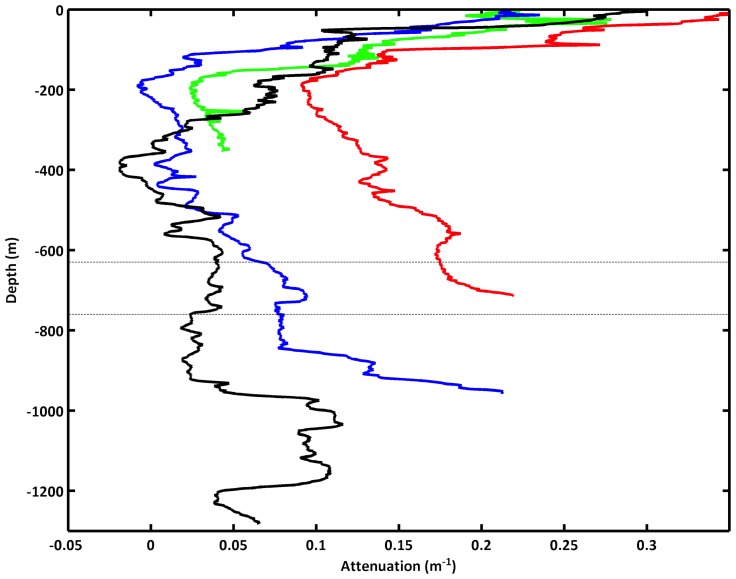
Light attenuation profiles (m^−1^) in the eastern branch of the Whittard canyon at 375 m (green), 750 m (red), 1000 m (blue) and 1320 m (black) water depth. Dashed lines (620–740 m) indicate depth of wall where high densities of suspension feeders were found.

Vertical profiles of beam attenuation (m^−1^), used as a proxy for suspended material concentration, revealed a strong benthic nepheloid layer (BNL) present and numerous intermediate nepheloid layers (INL) within the canyon (Figure 6). Overall beam attenuation (m^−1^) was highest in BNLs at 750 and 1000 m water depths. At 1000 m water depth, the BNL extended >100 m above the seabed, possibly associated with impinging MOW core found at this depth. A 200 m thick INL centred at 1100 m in the adjacent, deeper, vertical profile (water depth 1320 m) was likely associated with the detachment of the BNL. At the ROV dive site, the vertical profile of beam attenuation (m^−1^) indicated the highest values of suspended material extending from the seabed at 750 m to ∼500 m depth. INLs observed between 650–750 m water depth in the 1000 m and 1320 m profiles were also likely to be associated with the BNL found at the ROV site.

**Figure 7:**
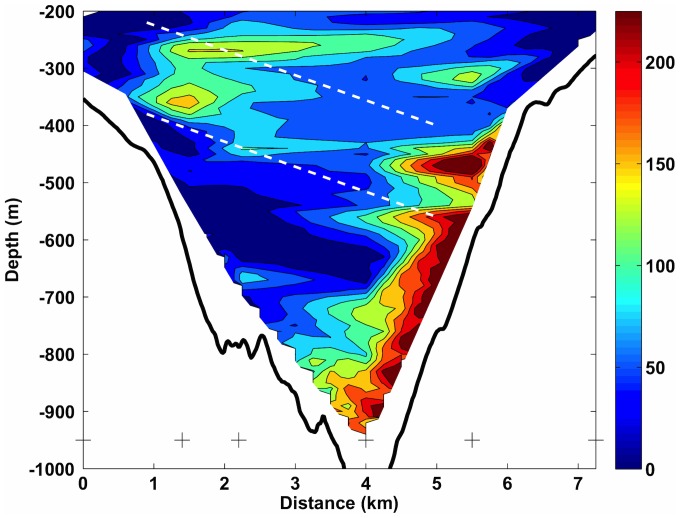
Transect of isopycnal displacement (absolute value in m) calculated from a 6–7 hr repeat transect across the canyon channel 4 km downstream of the ROV dive site. The southern side of the canyon (where the *A. excavata*-*N. pycnodonte* biotope was found) is on the right side of the figure. A scale is shown to the right, CTD locations by ‘x’ and the seabed by the black line. The parallel white lines across the transect indicate a possible beam of high isopycnal displacement associated with an internal wave emanating from the northern canyon wall.

The rough topographic nature of canyons is likely to result in dynamics of a complex nature where energy will be extracted from barotropic tides to baroclinic internal wave motion. A 6–7 hour repeat cross-canyon channel CTD transect ∼3 km downcanyon from the dive site suggested the presence of internal waves in the depth range 400–700 m (Figure 7). Internal waves generated at the barotropic semi-diurnal tidal period will propagate as a beam through the water column, periodically stretching and squashing the isopycnal surfaces at the depths where the internal wave energy is concentrated. This is highlighted in Figure 7 which shows the displacement of isopycnals based on the repeat CTD profiles at six locations across the canyon. At this time the upper 200 m of the water column was well mixed due to a severe storm a few days previously and is not shown. Maximum isopyncal displacement occurred close to the seabed at the dive site side of the canyon at ∼500 m and a secondary maximum was found at about 300 m on the opposite (northern) side. Relatively high isopycnal excursion occurred as a layer between these two maxima, as well as in a layer between 200–300 m which one might tentatively suggest emanated from the northern maximum. The band of high isopycnal excursion across the canyon between 300–500 m represented an angle (β) of ∼3 degrees from the horizontal. The buoyancy frequency (N), determined from vertical density profiles, together with an internal wave frequency (σ) appropriate for the semi-diurnal tide and Coriolis parameter (f), would suggest that internal waves would indeed propagate in beams 3 degrees from the horizontal (e.g. sin(β) = [(σ^2^−f^2^)/(N^2^−σ^2^) ]^1/2^, [21]). This perhaps suggests that internal waves may have been generated at the top of the north canyon wall, with subsequent cross canyon propagation to a location immediately above ROV dive site wall. In addition there is high displacement at the foot of the canyon which may be due to tidal modulation of any bottom density flow or of any MOW water located between 800–1000 m. This would cause a change in density from horizontal advection rather than vertical displacements.

The resolution of the CTD transect was such that repeat profiles were made at water depths of 500 and 1000 m respectively, spanning the depth range of the vertical wall. The high calculated displacements above 300 m may also be the result of an internal wave beam originating from the same source, propagating up and reflecting from the surface mixed layer, where there are high displacements immediately below the bottom of the surface mixed layer. Again the propagation angles are consistent although the interpretation is somewhat speculative. Overall the repeat CTD transect suggests that a significant amount of baroclinic energy exists within the upper/mid canyon region. A BNL was generated at the depth of high displacement on the southern canyon wall (500 m) and an INL at that depth was present within the canyon (Figure 6). It is likely therefore that sediment mobilisation at depths above the wall and its associated fauna is a persistent process within the canyon.

## Discussion

The *A. excavata* -*N. zibrowii* biotope has not been previously recognized and this habitat adds to the complexity and diversity of what is known from vertical faces at the continental margins. The group of six species associated with *A. excavata* or *N. zibrowii* also suggests a coherent assemblage responding to the same habitat cues. *Acesta excavata* has only previously been noted in such abundance from shallower depths on the sides of fjords [16]. High *N. zibrowii* cover has been observed in other canyon systems in the Bay of Biscay, but not in association with *A. excavata* [5]. The vertical face covered in *Lophelia pertusa* with occasional *A. excavata* described by Huvenne et al. [6] was at a deeper point of the Whittard Canyon system (1350 m). Other wall-associated assemblages seem likely, possibly including extensions of some of the rock assemblages identified by Howell et al. [17]. The source of debris in a gorge close to the Wyville-Thomson ridge has been suggested to be from barnacle populations (*Bathylasma hirsutum*) on adjacent rock walls [22].

The variation in densities and mean shell size imply that there are relatively small-scale vertical variations in resource supply. Larger and more numerous filter feeders with depth suggest that there is more food at greater depths, up to the point where the biomass is much lower. Observation of the suspended particulate matter (SPM) concentrations in the canyon (Figure 6) indicated a general increase in SPM at the depths occupied by the biotope, which was located within the permanent thermocline. The depth range of this large vertical density gradient is one where both tidal and residual current energy are often enhanced (e.g., [21]). The rough topography associated with the canyon branches will likely be a source (and sink) of baroclinic energy (internal waves) generated by the enhanced currents found at the margin, for both residual flows (e.g., [5]) and those of tidal origin (e.g., [13], [14]). The canyon topography itself will likely channel and focus internal waves, resulting in a complex spatial (vertical and horizontal) distribution of baroclinic energy within the canyon (e.g. Figure 7). For example, observations of periodic resuspension in Baltimore canyon were associated with the focussing of internal waves towards the canyon head, manifest as a cold-water bore propagating up the canyon [23]. Hotchkiss and Wunsch [24] also found focussing of internal wave energy in the bottom layer at the foot of the upper Hudson Canyon. It is plausible therefore that one might expect the highest suspended sediment concentrations to be found at the foot of the wall in the Whittard Canyon. The availability of suspended food resources may explain the increases in bivalve size and densities with depth, up to the point where disturbance may restrict the assemblage towards the base of the wall due to burial or abrasion.

The different assemblages found on rock walls may be caused by environmental preferences. Variations in assemblage structure seem unlikely simply to be an expression of depth alone. For example, *L. pertusa* has a depth range that completely overlaps the *A. excavata* -*N. zibrowii* association reported in the current study [25] and therefore might have been expected to be conspicuous on the surveyed habitat. The influences of substratum hardness, stability and texture on the settlement and survival of different species are unknown. Food quality and quantity may have a role to play in creating different assemblages. *Acesta excavata* has a high capacity for filter feeding and a low metabolic rate [26]. It is seems likely that, given heterogeneity in resource supply in the deep sea, other filter feeders may specialize on different resource availabilities to *A. excavata*. The greater filtering capacity of a bivalve may cope better with a highly episodic supply, while a coral may be more efficient with a more regular supply of food particles.

Although different species may have environmental preferences for particular wall habitats, it is not clear whether direct competition for resources has a role in structuring wall assemblages. *Acesta excavata*, *N. zibrowii* and a number of other taxa were positively correlated on a small scale, which would not support the hypothesis of competition for resources between species. If there are direct competitive interactions between different species found on walls, then the timescales may be long, given the apparent longevity of corals and *A. excavata* [16], [27]. The relatively large average size of *A. excavata* and the absence of clear size classes other than the mean size may indicate that the assemblage had one large recruitment event leading to *A. excavata* domination of the available rock surface. This could imply assemblages structured by space pre-emption. However, it is difficult to distinguish between a large recruitment at one point in time and a stable age structure topped up by a very low level of recruitment and a high survivorship. A greater number of growth rate estimates at different scales and in different topographic settings would help to develop a clearer basis for understanding the variation in wall assemblages.

Looking at the available bathymetry, it is clear that there may be many areas of near-vertical habitat in the canyons of the continental margin (e.g., [28]). As pointed out by Huvenne et al. [6], canyon walls may represent refuges from fishing-related disturbance for species that may be found across wider areas. The vertical habitats certainly contain structures that may act as nursery habitat for deep-sea fish and other mobile species. In our example, the three-dimensional microstructures created by the *A. excavata* -*N. zibrowii* assemblage provide diverse habitats for macrofaunal organisms, including the fish and mobile invertebrates visible in photographs. Comparative studies of canyon wall assemblages would provide excellent information about the supply and fate of organic matter at different scales along the continental margin.

## Methods

### Biological Observations

Observations were made using the deep-water ROV *Holland I* during a cruise on the RV *Celtic Explorer* to the Whittard Canyon system (cruise CE12006, Figure 1). ROV *Holland I* is a Quasar work class ROV rated to 3000 m. It is equipped with several video camera systems, an OE14-208 digital stills camera and has two robotic arms, a slurp sampler and storage boxes for collecting fauna. Material retained in the slurp chamber was sieved with a 0.5 mm sieve and used to verify identifications from photographs. ROV depth and position were established via a Sonardyne Ranger USBL beacon system. Cruise CE12006 was targeting vertical walls in canyon systems as potential sites of high biomass. Potential wall areas were identified from the available INFOMAR bathymetry by targeting regions with high slope. The INFOMAR (Integrated Mapping for the Sustainable Development of Ireland’s Marine Resource) project is a joint venture between the Geological Survey of Ireland and the Marine Institute and provides high quality bathymetry to 25 m resolution, interpolated from 100 m spaced point data (http://www.infomar.ie/data/). Suitable sites were dived with the ROV. On 16^th^ April 2012, a dive at 48.761°N, 10.461°W (cruise CE12006, Event number 12) encountered a vertical wall extending from 631 m depth to 780 m depth. On this wall we identified the presence of large numbers of *A. excavata* on the vertical faces. On reaching the top of the wall, the ROV was flown to near the bottom of the wall to repeat a vertical pass while taking still photographs. Poor weather prevented any further dives on or near this site during the cruise.

Individual, non-overlapping, photographs were treated as individual quadrats to collect information on size distributions and densities of *A. excavata*. Size and area estimates were made for each photograph using paired laser guidelines orientated at 90° to the camera’s focal plane and separated by 10 cm. Measurements, calibrated to that photograph’s laser guideline separation, were made in imageJ, an open source Java-based image processing progamme (http://rsb.info.nih.gov/ij/). Counts of *A. excavata* and *N. zibrowii* were converted to densities m^−2^. The attachment point of *A. excavata* shells to the substratum was taken as a basis for shell height measurements (the species has a straight dorsal margin). To minimize error, these measurements were only taken when the attachment point was clear and the shells were seen with a view of one valve or were seen in side view. *Neopycnodonte zibrowii* shells were similar in size to *A. excavata*, but the uneven nature of the oyster shells and the tendency for the edges of shells to overlap meant that shell sizes could not be confidently estimated. Quantile regressions were carried out using the quantreg package in R [29]. Overlapping photographs were mosaiced using check points added manually in the Hugin package (http://hugin.sourceforge.net/) to provide larger images of the assemblage. Organisms associated with the habitat were identified, where possible, from the images.

### Hydrographic Data

Sixty-six CTD casts were performed in two branches of the Whittard Canyon providing detailed hydrographic data. Vertical profiles of temperature, salinity, fluorescence and transmission were taken down to depths of 3150 m. Beam attenuation measurements were made using a 0.25 m path-length transmissometer (C-Star, WET Labs’) operating at 650 nm. Data were processed using Seabird data-processing software and MATLAB (Matworks, R2007a). Beam attenuation was calculated using
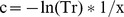
where x is the pathlength of the transmissometer and Tr is the transmittance output fromthe instrument expressed as



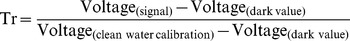



Voltage_(signal)_ is the output signal, Voltage_(dark value)_ is the dark offset for the instrument obtained by blocking the light path and Voltage_(clean water calibration)_ is the manufacturer’s supplied value for output in clean water.

Isopycnal displacements were calculated from a 6–7 hour repeat survey across the canyon close to the ROV dive site. To achieve this, the individual profiles were averaged over 20 m vertical bins to obtain individual vertical profiles of density and the density difference between the profile pair (ρ’). To calculate the isopycnal displacement (Z) at any depth, each pair of repeat profiles was averaged to form a mean vertical density profile and ρ’ was divided by the vertical density gradient of a range +/−40 m about each depth (dρ/dz),